# Supplementary figures and images for: Identification and characterization of a novel multi-stress responsive gene in Arabidopsis
Source: PLoS One. 2020 Dec 17;15(12):e0244030. doi: 10.1371/journal.pone.0244030 (PMC7746274; doi:10.1371/journal.pone.0244030)

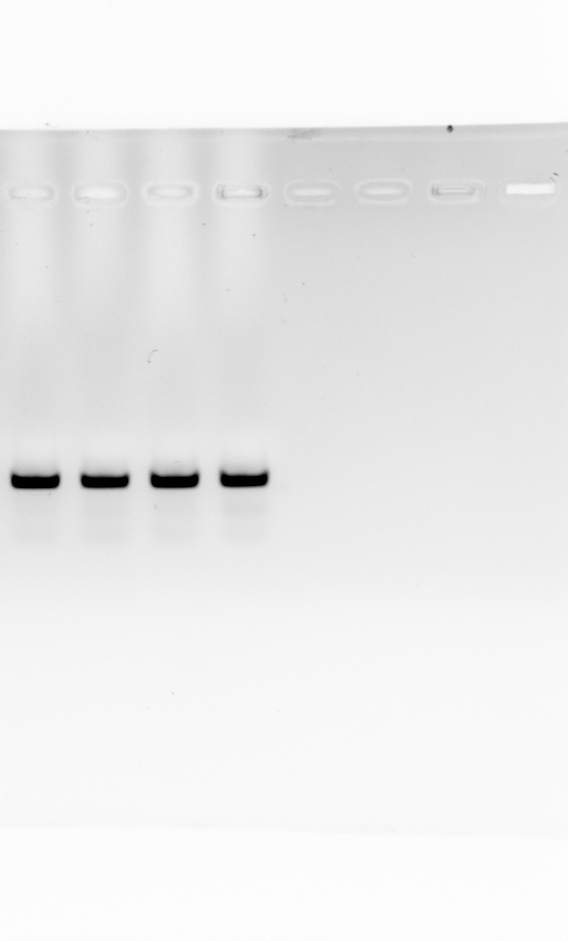

Supplement: S1 Fig — (TIFF) [file pone.0244030.s001.tiff]

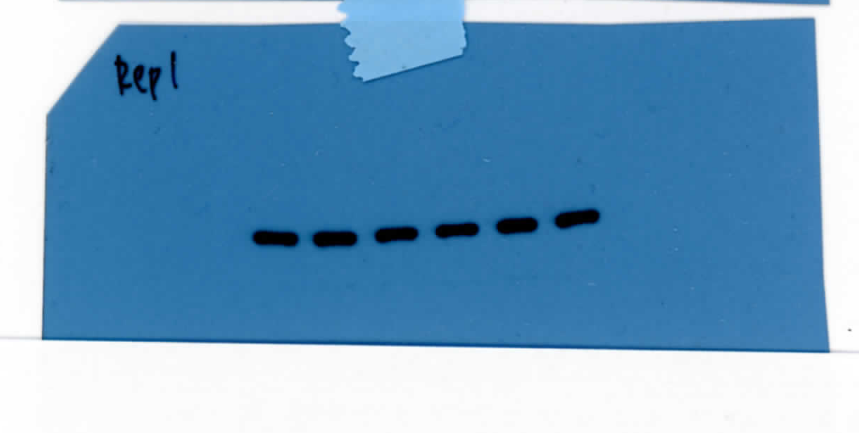

Supplement: S2 Fig — (TIFF) [file pone.0244030.s002.tiff]
