# Supplementary material for: Identification and characterization of a novel multi-stress responsive gene in Arabidopsis
Source: PLoS One. 2020 Dec 17;15(12):e0244030. doi: 10.1371/journal.pone.0244030 (PMC7746274; doi:10.1371/journal.pone.0244030)
Supplement: S1 Table — (DOCX) [file pone.0244030.s003.docx]

**Supplementary Table 1:**

**Primers used for RT-PCR**

| **Gene** | **Orientation** | **Sequence (5′ to 3′)** |
| --- | --- | --- |
| *AT4G18280* | Sense | AGTGGAAAAGGAAGGTGGCT |
|  | Antisense | ACTTCTTTTAGCGGTGTTGGC |

**Primers for *PGRPL1::GUS* cloning**

| **Gene** | **Orientation** | **Sequence (5′ to 3′)** |
| --- | --- | --- |
| *AT4G18280* | Sense | CTCTAGAGGATCCCCaaatttattccctcattattcattagtctctaatatttggttagatg |
|  | Antisense | AGGGACTGACCACCCgaatgccaaaaagaattaaaaatgcttttaagtaaaaaggag |

Uppercase letters = infusion/restriction enzyme sites, lowercase letters = genomic DNA sequence of *GPRL1*

**Primers used for qPCR**

| **Gene** | **Orientation** | **Sequence (5′ to 3′)** |
| --- | --- | --- |
| *AT4G18280* | Sense | GGTTCTGGCCATGGAAGTGG |
|  | Antisense | TTTAGCGGTGTTGGCTCCTC |
