# Supplementary material for: Identification and characterization of a novel multi-stress responsive gene in Arabidopsis
Source: PLoS One. 2020 Dec 17;15(12):e0244030. doi: 10.1371/journal.pone.0244030 (PMC7746274; doi:10.1371/journal.pone.0244030)
Supplement: S2 Table — (DOCX) [file pone.0244030.s004.docx]

**Supplementary Table 2:**

| **S. No.** | **Gene IDs** | **Names** |
| --- | --- | --- |
| 1 | AT1G64200 | VHA-E3 |
| 2 | AT5G64510 | TIN1 |
| 3 | AT5G59720 | HSP18.1 |
| 4 | AT5G58110 | AT5G58110 |
| 5 | AT5G52640 | HSP90-1 |
| 6 | AT5G48570 | FKBP65 |
| 7 | AT4G18280 | AT4G18280 |
| 8 | AT4G12400 | HOP3 |
| 9 | AT4G10250 | HSP22.0 |
| 10 | AT3G53230 | CDC48D |
| 11 | AT3G24100 | AT3G24100 |
| 12 | AT3G21670 | NPF6.4 |
| 13 | AT3G12580 | MED37C |
| 14 | AT2G37180 | PIP2-3 |
| 15 | AT2G33380 | PXG3 |
| 16 | AT2G29500 | HSP17.6B |
| 17 | AT2G16586 | AT2G16586 |
| 18 | AT1G72645 | AT1G72645 |
| 19 | AT1G07400 | HSP17.8 |
| 20 | AT5G24160 | SQE6 |
| 21 | None | None |
| 22 | AT1G71530 | AT1G71530 |
| 23 | AT1G73870 | COL7 |
| 24 | AT1G56600 | GOLS2 |
| 25 | AT3G55240 | AT3G55240 |
| 26 | AT3G11410 | PP2CA |
| 27 | AT4G30460 | AT4G30460 |
| 28 | AT2G20560 | AT2G20560 |
| 29 | AT5G51440 | HSP23.5 |
| 30 | AT3G24520 | HSFC1 |
| 31 | AT1G74310 | CLPB1 |
| 32 | AT5G12020 | HSP17.6 |
| 33 | AT1G75750 | GASA1 |
| 34 | AT5G19860 | AT5G19860 |
| 35 | AT3G48520 | CYP94B3 |
| 36 | AT2G32120 | HSP70-8 |
| 37 | AT1G60190 | PUB19 |
| 38 | AT4G37410 | CYP81F4 |
| 39 | AT5G03230 | AT5G03230 |
| 40 | None | None |
| 41 | AT1G10960 | FD1 |
| 42 | AT1G62510 | AT1G62510 |
| 43 | AT5G59220 | SAG113 |
| 44 | AT4G12560 | CPR1 |
| 45 | AT2G26690 | NPF6.2 |
| 46 | AT1G09140 | SR30 |
| 47 | AT5G66052 | AT5G66052 |
| 48 | AT5G25754 | AT5G25754 |
| 49 | AT3G26450 | AT3G26450 |
| 50 | AT1G03220 | AT1G03220 |
| 51 | AT5G55120 | VTC5 |
| 52 | AT4G27410 | RD26 |
| 53 | AT3G15010 | UBA2C |
| 54 | AT1G65980 | PRXIIB |
| 55 | AT1G30590 | AT1G30590 |
| 56 | AT4G37540 | LBD39 |
| 57 | None | None |
| 58 | AT1G59860 | HSP17.6A |
| 59 | AT4G28140 | ERF054 |
| 60 | AT1G65040 | HRD1B |
| 61 | None | None |
| 62 | AT2G36950 | HIPP05 |
| 63 | None | None |
| 64 | AT1G07350 | SR45A |
| 65 | AT2G38400 | AGT3 |
| 66 | AT1G70180 | AT1G70180 |
| 67 | AT3G53830 | AT3G53830 |
| 68 | AT1G66760 | DTX9 |
| 69 | AT5G47610 | ATL79 |
| 70 | AT5G66240 | AT5G66240 |
| 71 | AT1G16030 | HSP70-5 |
| 72 | AT1G20070 | AT1G20070 |
| 73 | AT2G26600 | AT2G26600 |
| 74 | AT1G67140 | SWEETIE |
| 75 | AT1G43160 | RAP2-6 |
| 76 | AT3G25840 | AT3G25840 |
| 77 | AT2G32530 | CSLB3 |
| 78 | AT2G37340 | RS2Z33 |
| 79 | AT1G78670 | GGH3 |
| 80 | AT1G50140 | AT1G50140 |
| 81 | AT4G34000 | ABF3 |
| 82 | AT3G55970 | JRG21 |
| 83 | AT5G02770 | MOS11 |
| 84 | AT2G38870 | AT2G38870 |
| 85 | AT3G13800 | AT3G13800 |
| 86 | AT3G14440 | NCED3 |
| 87 | AT3G07300 | AT3G07300 |
| 88 | AT5G62200 | ATS3B |
| 89 | AT5G11650 | AT5G11650 |
| 90 | AT5G25280 | AT5G25280 |
| 91 | AT4G14615 | AT4G14615 |
| 92 | AT5G57150 | AT5G57150 |
| 93 | AT1G30070 | AT1G30070 |
| 94 | AT4G03320 | TIC20-IV |
| 95 | AT1G16810 | AT1G16810 |
| 96 | AT1G69410 | ELF5A-3 |
| 97 | AT5G51890 | PER66 |
| 98 | AT4G10040 | CYTC-2 |
| 99 | AT1G67860 | AT1G67860 |
| 100 | AT5G61820 | AT5G61820 |
| 101 | AT2G29140 | APUM3 |
| 102 | AT4G23050 | AT4G23050 |
| 103 | AT4G02430 | SR34b |
| 104 | AT2G02130 | PDF2.3 |
| 105 | AT1G05340 | AT1G05340 |
| 106 | AT2G28400 | AT2G28400 |
| 107 | AT3G47420 | ATPS3 |
| 108 | AT1G73330 | ATDR4 |
| 109 | AT1G45201 | ATTLL1 |
| 110 | AT5G01600 | FER1 |
| 111 | AT1G10320 | AT1G10320 |
| 112 | AT5G48030 | GFA2 |
| 113 | AT5G05110 | CYS7 |
| 114 | AT2G25140 | CLPB4 |
| 115 | AT5G59440 | ZEU1 |
| 116 | AT3G46230 | HSP17.4A |
| 117 | AT3G62260 | AT3G62260 |
| 118 | AT2G37170 | PIP2B |
| 119 | AT3G29575 | AFP3 |
| 120 | AT4G13110 | AT4G13110 |
| 121 | AT5G09590 | HSP70-10 |
| 122 | AT3G07030 | AT3G07030 |
| 123 | AT2G30000 | AT2G30000 |
| 124 | AT5G21105 | AT5G21105 |
| 125 | AT2G26150 | HSFA2 |
| 126 | AT5G02020 | SIS |
| 127 | AT2G35940 | BLH1 |
| 128 | AT5G54170 | AT5G54170 |
| 129 | AT2G15790 | CYP40 |
| 130 | AT1G78790 | MHF2 |
| 131 | AT1G56170 | NFYC2 |
| 132 | AT1G27350 | AT1G27350 |
| 133 | AT1G05100 | MAPKKK18 |
| 134 | AT4G16280 | FCA |
| 135 | AT1G31600 | AT1G31600 |
| 136 | AT5G19875 | AT5G19875 |
| 137 | AT1G63530 | AT1G63530 |
| 138 | AT5G63130 | AT5G63130 |
| 139 | AT5G15090 | VDAC3 |
| 140 | None | None |
| 141 | AT2G24120 | RPOT3 |
| 142 | AT5G06340 | NUDT27 |
| 143 | AT1G56200 | emb1303 |
| 144 | AT3G20410 | CPK9 |
| 145 | AT3G53920 | SIGC |
| 146 | AT4G34710 | ADC2 |
| 147 | AT1G19660 | BBD2 |
| 148 | AT1G52890 | NAC019 |
| 149 | AT2G05540 | AT2G05540 |
| 150 | AT1G47510 | IP5P11 |
| 151 | AT5G48960 | AT5G48960 |
| 152 | AT5G26980 | SYP41 |
| 153 | AT2G46270 | GBF3 |
| 154 | AT4G16680 | AT4G16680 |
| 155 | AT5G38150 | PMI15 |
| 156 | AT3G41762 | AT3G41762 |
| 157 | AT2G20880 | ERF053 |
| 158 | AT1G01720 | NAC002 |
| 159 | AT5G41400 | AT5G41400 |
| 160 | AT1G55920 | SAT1 |
| 161 | AT3G47910 | AT3G47910 |
| 162 | None | None |
| 163 | AT1G76590 | AT1G76590 |
| 164 | AT2G38530 | LTP2 |
| 165 | AT1G64500 | AT1G64500 |
| 166 | AT3G47680 | AT3G47680 |
| 167 | AT3G15500 | NAC055 |
| 168 | AT2G36080 | ARF31 |
| 169 | AT3G05690 | UNE8 |
| 170 | AT3G27280 | PHB4 |
| 171 | AT1G70900 | AT1G70900 |
| 172 | AT2G21560 | AT2G21560 |
| 173 | AT1G32870 | ANAC13 |
| 174 | AT1G21580 | AT1G21580 |
| 175 | AT1G30230 | AT1G30230 |
| 176 | AT2G31280 | CPUORF7 |
| 177 | AT4G36648 | AT4G36648 |
| 178 | AT2G40360 | BOP1 |
| 179 | AT1G26580 | AT1G26580 |
| 180 | AT2G18440 | GUT15 |
| 181 | AT1G64720 | CP5 |
| 182 | AT3G01820 | AT3G01820 |
| 183 | AT1G76130 | AMY2 |
| 184 | AT3G19100 | CRK2 |
| 185 | AT1G70300 | POT6 |
| 186 | AT3G53970 | AT3G53970 |
| 187 | AT2G04160 | AIR3 |
| 188 | AT1G77450 | NAC032 |
| 189 | AT1G73750 | AT1G73750 |
| 190 | AT1G07150 | MAPKKK13 |
| 191 | AT1G77180 | SKIP |
| 192 | AT3G12050 | AT3G12050 |
| 193 | AT5G46410 | SSP4 |
| 194 | AT1G75670 | AT1G75670 |
| 195 | AT5G45140 | NRPC2 |
| 196 | AT3G19030 | AT3G19030 |
| 197 | AT3G63010 | GID1B |
| 198 | AT2G25970 | AT2G25970 |
| 199 | AT3G10030 | AT3G10030 |
| 200 | AT4G36690 | U2AF65A |
| 201 | AT4G38060 | AT4G38060 |
| 202 | AT1G72800 | AT1G72800 |
| 203 | AT5G55060 | AT5G55060 |
| 204 | AT1G52560 | HSP26.5 |
| 205 | AT5G10300 | HNL |
| 206 | AT1G69750 | COX19-2 |
| 207 | AT1G61620 | CSU1 |
| 208 | AT4G26080 | ABI1 |
| 209 | AT4G32440 | AT4G32440 |
| 210 | AT4G01400 | AT4G01400 |
| 211 | AT1G08700 | PS1 |
| 212 | AT2G04690 | AT2G04690 |
| 213 | AT2G26800 | HMGCL |
| 214 | AT1G54050 | HSP17.4B |
| 215 | AT4G40060 | ATHB-16 |
| 216 | AT3G16640 | TCTP1 |
| 217 | AT3G12300 | AT3G12300 |
| 218 | AT1G56145 | AT1G56145 |
| 219 | AT5G34850 | PAP26 |
| 220 | AT4G04620 | ATG8B |
| 221 | AT5G59780 | MYB59 |
| 222 | AT5G64230 | AT5G64230 |
| 223 | AT4G03030 | OR23 |
| 224 | AT4G23670 | AT4G23670 |
| 225 | AT1G54100 | ALDH7B4 |
| 226 | AT5G57565 | AT5G57565 |
| 227 | AT4G01120 | GBF2 |
| 228 | AT2G42330 | STIPL2 |
| 229 | AT3G61630 | CRF6 |
| 230 | AT4G15910 | ATDI21 |
| 231 | AT4G06634 | YY1 |
| 232 | AT2G31350 | GLX2-5 |
| 233 | AT5G13220 | JAZ10 |
| 234 | AT1G80160 | AT1G80160 |
| 235 | AT2G44710 | AT2G44710 |
| 236 | AT2G46240 | BAG6 |
| 237 | AT4G11240 | TOPP6 |
| 238 | AT1G09530 | PIF3 |
| 239 | AT5G27660 | AT5G27660 |
| 240 | AT2G15080 | AtRLP19 |
| 241 | AT2G39580 | AT2G39580 |
| 242 | AT5G13820 | TRP4 |
| 243 | AT1G63490 | AT1G63490 |
| 244 | AT2G05440 | ATGRP9 |
| 245 | AT5G03730 | CTR1 |
| 246 | AT4G37180 | HHO5 |
| 247 | AT3G50340 | AT3G50340 |
| 248 | AT3G54500 | AT3G54500 |
| 249 | AT2G27500 | AT2G27500 |
| 250 | AT1G53320 | TULP7 |
| 251 | AT2G29400 | TOPP1 |
| 252 | AT1G73150 | GTE3 |
| 253 | AT3G20470 | GRP5 |
| 254 | AT4G28290 | AT4G28290 |
| 255 | AT4G32850 | nPAP |
| 256 | AT1G64660 | MGL |
| 257 | AT1G05562 | AT1G05562 |
| 258 | AT5G56850 | AT5G56850 |
| 259 | AT4G26110 | NAP1;1 |
| 260 | AT3G18215 | AT3G18215 |
| 261 | AT2G45920 | PUB37 |
| 262 | AT3G19580 | AZF2 |
| 263 | AT1G77885 | AT1G77885 |
| 264 | AT1G54770 | AT1G54770 |
| 265 | AT3G29390 | RIK |
| 266 | AT5G63370 | CDKG1 |
| 267 | AT3G60690 | AT3G60690 |
| 268 | AT2G28110 | IRX7 |
| 269 | AT3G62290 | ARFA1E |
| 270 | AT5G43150 | AT5G43150 |
| 271 | AT5G13800 | PPH |
| 272 | None | None |
| 273 | AT1G71696 | SOL1 |
| 274 | AT1G15380 | AT1G15380 |
| 275 | AT4G03430 | STA1 |
| 276 | AT2G37150 | AT2G37150 |
| 277 | AT4G31820 | NPY1 |
| 278 | AT1G79520 | AT1G79520 |
| 279 | AT2G17787 | AT2G17787 |
| 280 | AT5G35210 | AT5G35210 |
| 281 | AT5G03560 | AT5G03560 |
| 282 | AT3G50670 | RNU1 |
| 283 | AT1G69490 | NAC029 |
| 284 | AT2G39310 | JAL22 |
| 285 | AT1G78070 | AT1G78070 |
| 286 | AT4G16780 | HAT4 |
| 287 | AT4G27860 | AT4G27860 |
| 288 | AT4G39270 | AT4G39270 |
| 289 | AT1G08310 | AT1G08310 |
| 290 | AT1G02850 | BGLU11 |
| 291 | AT5G49665 | WAV3 |
| 292 | AT3G56080 | AT3G56080 |
| 293 | AT3G57540 | REM4.1 |
| 294 | AT5G26600 | CpNIFS3 |
| 295 | AT5G15270 | AT5G15270 |
| 296 | AT1G20880 | AT1G20880 |
| 297 | AT2G22470 | AGP2 |
| 298 | AT5G54470 | AT5G54470 |
| 299 | AT5G58470 | TAF15B |
| 300 | AT1G34110 | AT1G34110 |
| 301 | AT1G08230 | GAT1 |
| 302 | AT5G02840 | RVE4 |
| 303 | AT1G50260 | NTMC2TYPE5.1 |
| 304 | AT1G30500 | NFYA7 |
| 305 | AT2G04240 | XERICO |
| 306 | AT1G61970 | AT1G61970 |
| 307 | AT3G51500 | AT3G51500 |
| 308 | AT1G20920 | RH42 |
| 309 | AT2G41060 | UBA2B |
| 310 | AT1G69260 | AFP1 |
| 311 | AT1G72770 | HAB1 |
| 312 | AT2G01008 | AT2G01008 |
| 313 | AT2G45960 | PIP1B |
| 314 | AT2G15000 | AT2G15000 |
| 315 | AT2G29300 | AT2G29300 |
| 316 | AT3G06400 | CHR11 |
| 317 | AT3G61890 | ATHB-12 |
| 318 | AT3G19670 | PRP40B |
| 319 | AT2G46680 | ATHB-7 |
| 320 | AT1G17020 | SRG1 |
| 321 | AT5G19110 | AT5G19110 |
| 322 | AT2G21320 | BBX18 |
| 323 | AT4G12760 | AT4G12760 |
| 324 | AT1G32530 | MIP1 |
| 325 | AT1G06570 | HPD |
| 326 | AT1G22640 | MYB3 |
| 327 | AT2G36792 | AT2G36792 |
| 328 | AT3G07610 | IBM1 |
| 329 | AT1G56280 | ATDI19 |
| 330 | AT3G02480 | AT3G02480 |
| 331 | AT1G09500 | AT1G09500 |
| 332 | AT5G12840 | NFYA1 |
| 333 | AT3G23790 | AAE16 |
| 334 | AT1G02220 | NAC003 |
| 335 | AT3G28210 | SAP12 |
| 336 | AT3G60410 | AT3G60410 |
| 337 | AT2G36780 | UGT73C3 |
| 338 | AT4G36900 | RAP2-10 |
| 339 | AT2G02390 | ATGSTZ1 |
| 340 | AT2G47180 | GOLS1 |
| 341 | AT2G36800 | UGT73C5 |
| 342 | AT2G47060 | AT2G47060 |
| 343 | AT2G38540 | LTP1 |
| 344 | AT5G54080 | HGO |
| 345 | AT5G64390 | HEN4 |
| 346 | AT3G27220 | AT3G27220 |
| 347 | AT1G48410 | AGO1 |
| 348 | AT3G26920 | AT3G26920 |
| 349 | AT4G00710 | BSK3 |
| 350 | AT4G21560 | VPS28-1 |
| 351 | AT5G51410 | AT5G51410 |
| 352 | AT3G26910 | AT3G26910 |
| 353 | AT5G64170 | LNK1 |
| 354 | AT5G46470 | RPS6 |
| 355 | AT1G55310 | SR33 |
| 356 | AT4G33420 | AT4G33420 |
| 357 | AT5G63970 | RGLG3 |
| 358 | AT3G57390 | AGL18 |
| 359 | AT1G15240 | AT1G15240 |
| 360 | AT1G29357 | AT1G29357 |
| 361 | AT5G04540 | MTM2 |
| 362 | AT5G15540 | EMB2773 |
| 363 | AT1G01210 | AT1G01210 |
| 364 | AT4G38090 | AT4G38090 |
| 365 | AT1G10050 | AT1G10050 |
| 366 | AT3G07350 | AT3G07350 |
| 367 | AT5G13330 | ERF113 |
| 368 | AT3G28690 | AT3G28690 |
| 369 | AT3G02150 | TCP13 |
| 370 | AT5G15500 | AT5G15500 |
| 371 | AT3G26510 | AT3G26510 |
| 372 | AT3G27170 | CLC-B |
| 373 | AT3G12520 | SULTR4;2 |
| 374 | AT5G65380 | DTX27 |
| 375 | AT3G09560 | PAH1 |
| 376 | AT1G42540 | GLR3.3 |
| 377 | AT1G73010 | PS2 |
| 378 | AT5G45800 | MEE62 |
| 379 | AT2G24540 | AFR |
| 380 | AT1G20640 | NLP4 |
| 381 | AT2G24520 | AHA5 |
| 382 | AT5G22860 | AT5G22860 |
| 383 | AT3G56260 | AT3G56260 |
| 384 | AT1G03550 | SCAMP2 |
| 385 | AT5G22720 | AT5G22720 |
| 386 | AT2G20720 | AT2G20720 |
| 387 | AT5G35450 | RPP8L3 |
| 388 | AT2G39830 | DAR2 |
| 389 | AT4G18140 | SSP4b |
| 390 | AT1G33420 | AT1G33420 |
| 391 | AT5G53180 | ATPTB2 |
| 392 | AT4G14410 | BHLH104 |
| 393 | AT3G46660 | UGT76E12 |
| 394 | AT4G26230 | RPL31B |
| 395 | AT2G39340 | SAC3A |
| 396 | AT1G22370 | UGT85A5 |
| 397 | AT3G62000 | AT3G62000 |
| 398 | AT5G61560 | AT5G61560 |
| 399 | AT5G04440 | AT5G04440 |
| 400 | AT5G03345 | AT5G03345 |
| 401 | AT1G50970 | AT1G50970 |
| 402 | AT1G50500 | HIT1 |
| 403 | AT1G45249 | ABF2 |
| 404 | AT1G42430 | AT1G42430 |
| 405 | AT1G34370 | STOP1 |
| 406 | AT1G33290 | AT1G33290 |
| 407 | AT1G31540 | AT1G31540 |
| 408 | AT1G30755 | AT1G30755 |
| 409 | AT1G29060 | AT1G29060 |
| 410 | AT5G67290 | AT5G67290 |
| 411 | AT5G67030 | ZEP |
| 412 | AT5G65685 | AT5G65685 |
| 413 | AT5G63710 | AT5G63710 |
| 414 | AT5G62050 | OXA1 |
| 415 | AT5G60990 | RH10 |
| 416 | AT5G59710 | VIP2 |
| 417 | AT5G58350 | WNK4 |
| 418 | AT5G57050 | ABI2 |
| 419 | AT5G52530 | AT5G52530 |
| 420 | AT5G50160 | FRO8 |
| 421 | AT5G48620 | RPP8L4 |
| 422 | AT5G41960 | AT5G41960 |
| 423 | AT1G22860 | VPS3 |
| 424 | AT1G22430 | AT1G22430 |
| 425 | AT5G25570 | AT5G25570 |
| 426 | AT1G21920 | AT1G21920 |
| 427 | AT5G22510 | INVE |
| 428 | AT5G19660 | SBT6.1 |
| 429 | AT5G19430 | AT5G19430 |
| 430 | AT5G13760 | AT5G13760 |
| 431 | AT5G13550 | SULTR4;1 |
| 432 | AT5G10070 | AT5G10070 |
| 433 | AT5G07740 | AT5G07740 |
| 434 | AT5G06350 | AT5G06350 |
| 435 | AT5G06280 | AT5G06280 |
| 436 | AT5G05540 | SDN2 |
| 437 | AT5G03500 | AT5G03500 |
| 438 | AT5G01950 | AT5G01950 |
| 439 | AT5G01220 | SQD2 |
| 440 | AT4G18400 | AT4G18400 |
| 441 | AT4G13040 | AT4G13040 |
| 442 | AT4G08480 | MEKK2 |
| 443 | AT3G63310 | BIL4 |
| 444 | AT3G62240 | AT3G62240 |
| 445 | AT3G61700 | AT3G61700 |
| 446 | AT3G61420 | TFB1-3 |
| 447 | AT3G61320 | AT3G61320 |
| 448 | AT3G61010 | AT3G61010 |
| 449 | AT1G15180 | DTX13 |
| 450 | AT3G53500 | RS2Z32 |
| 451 | AT3G51950 | AT3G51950 |
| 452 | AT3G49430 | SR34A |
| 453 | AT3G46450 | AT3G46450 |
| 454 | AT3G43240 | ARID4 |
| 455 | AT3G33530 | AT3G33530 |
| 456 | AT3G25570 | SAMDC3 |
| 457 | AT3G20270 | AT3G20270 |
| 458 | AT1G12680 | PEPKR2 |
| 459 | AT3G14830 | AT3G14830 |
| 460 | AT3G12140 | AT3G12140 |
| 461 | AT3G11964 | RRP5 |
| 462 | AT3G09410 | PAE5 |
| 463 | AT3G06500 | INVC |
| 464 | AT3G06190 | BPM2 |
| 465 | AT3G05060 | NOP5-2 |
| 466 | AT1G10890 | AT1G10890 |
| 467 | AT2G46830 | CCA1 |
| 468 | AT2G46420 | AT2G46420 |
| 469 | AT2G43920 | HOL2 |
| 470 | AT2G43330 | INT1 |
| 471 | AT2G43020 | PAO2 |
| 472 | AT2G42270 | BRR2B |
| 473 | AT1G01790 | KEA1 |
| 474 | AT2G40270 | AT2G40270 |
| 475 | AT2G36720 | AT2G36720 |
| 476 | AT2G32040 | AT2G32040 |
| 477 | AT2G25590 | AT2G25590 |
| 478 | AT1G01710 | AT1G01710 |
| 479 | AT2G15890 | MEE14 |
| 480 | AT2G13650 | GONST1 |
| 481 | AT2G03640 | AT2G03640 |
| 482 | AT1G01650 | SPPL4 |
| 483 | AT2G01450 | MPK17 |
| 484 | AT1G80640 | AT1G80640 |
| 485 | AT1G79270 | ECT8 |
| 486 | AT1G75850 | VPS35B |
| 487 | AT1G73480 | AT1G73480 |
| 488 | AT1G71220 | EBS1 |
| 489 | AT1G70570 | AT1G70570 |
| 490 | AT1G68580 | AT1G68580 |
| 491 | AT1G68020 | TPS6 |
| 492 | AT1G67325 | AT1G67325 |
| 493 | AT1G04830 | AT1G04830 |
| 494 | AT1G61380 | SD129 |
| 495 | AT1G60270 | BGLU6 |
| 496 | AT1G58200 | MSL3 |
| 497 | AT1G53390 | ABCG24 |
| 498 | AT1G53310 | PPC1 |
| 499 | AT5G63460 | AT5G63460 |
| 500 | AT5G63320 | GTE10 |
| 501 | AT5G63050 | EMB2759 |
| 502 | AT5G60600 | ISPG |
| 503 | AT5G60120 | TOE2 |
| 504 | AT5G57340 | AT5G57340 |
| 505 | AT5G54300 | AT5G54300 |
| 506 | AT5G53160 | PYL8 |
| 507 | AT5G48160 | OBE2 |
| 508 | AT5G47430 | AT5G47430 |
| 509 | AT5G45410 | AT5G45410 |
| 510 | AT5G44750 | REV1 |
| 511 | AT5G43180 | AT5G43180 |
| 512 | AT5G38470 | RAD23D |
| 513 | AT5G28050 | AT5G28050 |
| 514 | AT5G25770 | AT5G25770 |
| 515 | AT5G23450 | ATLCBK1 |
| 516 | AT5G22875 | AT5G22875 |
| 517 | AT5G22770 | ALPHA-ADR |
| 518 | AT5G19030 | AT5G19030 |
| 519 | AT5G16660 | AT5G16660 |
| 520 | AT5G15610 | AT5G15610 |
| 521 | AT5G13750 | ZIFL1 |
| 522 | AT5G05690 | CYP90A1 |
| 523 | AT5G04250 | AT5G04250 |
| 524 | AT5G03240 | UBQ3 |
| 525 | AT4G38810 | AT4G38810 |
| 526 | AT4G36730 | GBF1 |
| 527 | AT4G33300 | ADR1-L1 |
| 528 | AT4G28260 | AT4G28260 |
| 529 | AT4G26965 | AT4G26965 |
| 530 | AT4G24800 | AT4G24800 |
| 531 | AT4G24230 | ACBP3 |
| 532 | AT4G23620 | AT4G23620 |
| 533 | AT4G21105 | AT4G21105 |
| 534 | AT4G18020 | APRR2 |
| 535 | AT4G18010 | IP5P2 |
| 536 | AT4G15550 | UGT75D1 |
| 537 | AT4G15180 | ATXR3 |
| 538 | AT4G13100 | AT4G13100 |
| 539 | AT4G08980 | FBW2 |
| 540 | AT4G02725 | AT4G02725 |
| 541 | AT3G62650 | AT3G62650 |
| 542 | AT3G61220 | AT3G61220 |
| 543 | AT3G57470 | AT3G57470 |
| 544 | AT3G57050 | CBL |
| 545 | AT3G56310 | AGAL3 |
| 546 | AT3G55880 | SUE4 |
| 547 | AT3G53090 | UPL7 |
| 548 | AT3G51830 | SAC8 |
| 549 | AT3G51630 | WNK5 |
| 550 | AT3G49590 | AT3G49590 |
| 551 | AT3G44630 | AT3G44630 |
| 552 | AT3G28740 | CYP81D11 |
| 553 | AT3G26690 | NUDT13 |
| 554 | AT3G24500 | MBF1C |
| 555 | AT3G17790 | PAP17 |
| 556 | AT3G14810 | MSL5 |
| 557 | AT3G14420 | GLO1 |
| 558 | AT3G14350 | SRF7 |
| 559 | AT3G13580 | RPL7D |
| 560 | AT3G13110 | SAT3 |
| 561 | AT3G10300 | AT3G10300 |
| 562 | AT3G08690 | UBC11 |
| 563 | AT3G04810 | NEK2 |
| 564 | AT2G44065 | AT2G44065 |
| 565 | AT2G39760 | BPM3 |
| 566 | AT2G29650 | ANTR1 |
| 567 | AT2G26670 | HO1 |
| 568 | AT2G23450 | WAKL14 |
| 569 | AT2G17500 | PILS5 |
| 570 | AT2G16930 | AT2G16930 |
| 571 | AT2G15580 | AT2G15580 |
| 572 | AT1G72320 | APUM23 |
| 573 | AT1G68660 | CPLS1 |
| 574 | AT1G64860 | SIGA |
| 575 | None | None |
| 576 | AT1G64230 | UBC28 |
| 577 | AT1G64050 | AT1G64050 |
| 578 | AT1G61660 | BHLH112 |
| 579 | AT1G61360 | AT1G61360 |
| 580 | AT1G61150 | GID8 |
| 581 | AT1G56612 | AT1G56612 |
| 582 | AT1G55325 | GCT |
| 583 | AT1G54920 | AT1G54920 |
| 584 | AT1G54390 | ING2 |
| 585 | AT1G53380 | AT1G53380 |
| 586 | AT1G53090 | SPA4 |
| 587 | AT1G50030 | TOR |
| 588 | AT1G35340 | AT1G35340 |
| 589 | AT1G33970 | IAN9 |
| 590 | AT1G33110 | DTX21 |
| 591 | AT1G33050 | AT1G33050 |
| 592 | AT1G28660 | AT1G28660 |
| 593 | AT1G22930 | AT1G22930 |
| 594 | AT1G21350 | AT1G21350 |
| 595 | AT1G11840 | ATGLX1 |
| 596 | AT1G11280 | AT1G11280 |
| 597 | AT1G10820 | AT1G10820 |
| 598 | AT1G09060 | AT1G09060 |
| 599 | AT1G07590 | AT1G07590 |
| 600 | AT1G06630 | AT1G06630 |
| 601 | AT1G05890 | ARI5 |
| 602 | AT1G05560 | UGT1 |
| 603 | AT3G56940 | CRD1 |
| 604 | AT1G20620 | CAT3 |
| 605 | AT4G35090 | CAT2 |
| 606 | AT3G09350 | Fes1A |
| 607 | AT5G05410 | DREB2A |
| 608 | AT4G05050 | UBQ11 |
| 609 | AT3G23920 | BAM1 |
| 610 | AT5G16110 | AT5G16110 |
| 611 | AT3G14200 | AT3G14200 |
| 612 | AT3G55770 | AT3G55770 |
| 613 | AT2G46220 | AT2G46220 |
| 614 | AT3G48690 | CXE12 |
| 615 | AT1G67195 | MIR414 |
| 616 | AT1G64750 | ATDSS1(I) |
| 617 | AT5G62020 | HSFB2A |
| 618 | AT2G27530 | RPL10AB |
| 619 | AT2G40000 | HSPRO2 |
| 620 | AT1G15340 | MBD10 |
| 621 | AT5G64840 | ABCF5 |
| 622 | AT3G47340 | ASN1 |
| 623 | AT3G23000 | CIPK7 |
| 624 | AT2G33590 | AT2G33590 |
| 625 | AT3G09600 | RVE8 |
| 626 | AT2G41160 | RBL18 |
| 627 | AT1G01490 | AT1G01490 |
| 628 | AT3G58490 | LPPD |
| 629 | AT1G65370 | AT1G65370 |
| 630 | AT5G15960 | KIN1 |
| 631 | AT5G56100 | AT5G56100 |
| 632 | AT4G30490 | AT4G30490 |
| 633 | AT4G17730 | SYP23 |
| 634 | AT5G62190 | RH7 |
| 635 | AT3G05165 | AT3G05165 |
| 636 | AT1G80130 | AT1G80130 |
| 637 | AT2G22088 | AT2G22088 |
| 638 | AT2G36900 | MEMB11 |
| 639 | AT3G52060 | AT3G52060 |
| 640 | AT5G22000 | RHF2A |
| 641 | AT5G24670 | AT5G24670 |
| 642 | AT5G24800 | BZIP9 |
| 643 | AT1G17710 | AT1G17710 |
| 644 | AT1G10585 | BHLH167 |
| 645 | AT4G28300 | AT4G28300 |
| 646 | AT1G29970 | RPL18AA |
| 647 | AT5G13100 | AT5G13100 |
| 648 | AT1G11400 | PYM |
| 649 | AT4G28390 | AAC3 |
| 650 | AT1G66500 | PCFS1 |
| 651 | AT3G13040 | PHL6 |
| 652 | AT2G16720 | MYB7 |
| 653 | AT5G19180 | ECR1 |
| 654 | AT3G29185 | AT3G29185 |
| 655 | AT5G46840 | AT5G46840 |
| 656 | AT1G79790 | AT1G79790 |
| 657 | AT5G01820 | CIPK14 |
| 658 | AT5G25220 | KNAT3 |
| 659 | AT4G16330 | AT4G16330 |
| 660 | AT2G26430 | CYCL1-1 |
| 661 | AT1G05840 | AT1G05840 |
| 662 | AT5G45630 | AT5G45630 |
| 663 | AT2G44210 | AT2G44210 |
| 664 | None | None |
| 665 | AT4G15248 | MIP1A |
| 666 | AT1G13820 | AT1G13820 |
| 667 | AT3G12080 | emb2738 |
| 668 | AT1G52565 | AT1G52565 |
| 669 | AT3G14010 | CID4 |
| 670 | AT2G22660 | GRDP1 |
| 671 | AT4G14910 | HISN5B |
| 672 | AT1G70620 | AT1G70620 |
| 673 | AT4G25690 | AT4G25690 |
| 674 | AT1G53170 | ERF8 |
| 675 | AT3G16800 | AT3G16800 |
| 676 | AT2G22300 | CAMTA3 |
| 677 | AT5G25270 | AT5G25270 |
| 678 | AT3G11560 | AT3G11560 |
| 679 | AT2G01320 | AT2G01320 |
| 680 | AT2G04940 | AT2G04940 |
| 681 | AT1G73390 | AT1G73390 |
| 682 | AT1G29400 | ML5 |
| 683 | AT3G59950 | ATG4B |
| 684 | AT2G26580 | YAB5 |
| 685 | AT3G04930 | AT3G04930 |
| 686 | AT1G70940 | PIN3 |
| 687 | AT3G62770 | ATG18A |
| 688 | AT1G69760 | AT1G69760 |
| 689 | AT4G34100 | SUD1 |
| 690 | AT2G15020 | AT2G15020 |
| 691 | AT2G38880 | NF-YB1 |
| 692 | AT4G25720 | QCT |
| 693 | AT3G03310 | LCAT3 |
| 694 | AT4G35780 | STY17 |
| 695 | AT4G26910 | AT4G26910 |
| 696 | AT1G17145 | DA2L |
| 697 | AT3G47990 | SIS3 |
| 698 | AT1G36070 | AT1G36070 |
| 699 | AT1G64440 | UGE4 |
| 700 | AT1G32928 | AT1G32928 |
| 701 | AT5G09330 | NAC082 |
| 702 | AT5G65210 | TGA1 |
| 703 | AT1G10095 | AT1G10095 |
| 704 | AT5G19390 | ROPGAP7 |
| 705 | AT4G34131 | UGT73B3 |
| 706 | AT5G51290 | CERK |
| 707 | AT1G22180 | AT1G22180 |
| 708 | AT2G45150 | CDS4 |
| 709 | AT1G15110 | AT1G15110 |
| 710 | AT5G10625 | FLP2 |
| 711 | AT5G27950 | KIN14U |
| 712 | AT2G39280 | AT2G39280 |
| 713 | AT3G58640 | AT3G58640 |
| 714 | AT3G53100 | AT3G53100 |
| 715 | AT1G09570 | PHYA |
| 716 | AT1G30400 | ABCC1 |
| 717 | AT1G09010 | EBM |
| 718 | AT2G42890 | ML2 |
| 719 | AT4G32030 | AT4G32030 |
| 720 | AT5G04040 | SDP1 |
| 721 | AT2G38820 | AT2G38820 |
| 722 | AT5G19300 | AT5G19300 |
| 723 | AT1G71960 | ABCG25 |
| 724 | AT3G61710 | ATG6 |
| 725 | AT3G57300 | INO80 |
| 726 | AT3G04070 | NAC047 |
| 727 | AT2G36810 | SGR6 |
| 728 | AT1G05960 | AT1G05960 |
| 729 | AT5G43620 | PCFS5 |
| 730 | AT1G05790 | AT1G05790 |
| 731 | AT2G30070 | POT1 |
| 732 | AT4G32250 | AT4G32250 |
| 733 | None | None |
| 734 | AT4G32360 | MFDR |
| 735 | AT4G27820 | BGLU9 |
| 736 | AT3G17070 | PER29 |
| 737 | AT1G05570 | CALS1 |
| 738 | AT2G41210 | PIP5K5 |
| 739 | AT3G47730 | ABCA2 |
| 740 | AT4G36630 | EMB2754 |
| 741 | AT5G15020 | SNL2 |
| 742 | AT5G46490 | AT5G46490 |
| 743 | AT3G55480 | PAT2 |
| 744 | AT1G80260 | emb1427 |
| 745 | AT2G47770 | TSPO |
| 746 | AT2G44798 | AT2G44798 |
| 747 | AT4G02760 | AT4G02760 |
| 748 | AT4G38120 | AT4G38120 |
| 749 | AT3G14590 | NTMC2TYPE6.2 |
| 750 | AT5G17890 | DAR4 |
| 751 | AT1G50730 | AT1G50730 |
| 752 | AT5G45310 | AT5G45310 |
| 753 | AT2G45880 | BAM7 |
| 754 | AT2G31970 | RAD50 |
| 755 | AT3G44680 | HDA9 |
| 756 | AT1G17580 | XI-1 |
| 757 | AT1G55350 | DEK1 |
| 758 | AT1G33410 | SAR1 |
| 759 | AT4G32730 | PC-MYB1 |
| 760 | AT2G26780 | AT2G26780 |
