# Supplementary material for: Identification and characterization of a novel multi-stress responsive gene in Arabidopsis
Source: PLoS One. 2020 Dec 17;15(12):e0244030. doi: 10.1371/journal.pone.0244030 (PMC7746274; doi:10.1371/journal.pone.0244030)
Supplement: Attachment — Submitted filename: Response to Reviewer R1.docx [file pone.0244030.s006.docx]

**Response to Reviewers**

Reviewer #1

Line 58 full form of GRPs should be mentioned (when it is mentioned first time)

We thank Reviewer 1 for highlighting this; the full form of GRPs is now mentioned in line 58.

Ilalics for gene names

All gene names have been italicized.

Line 65-67 may be deleted

Lines 65-67 have been removed.

Method of calculation for qRT-PCR should be included in the expression analysis section.

The qPCR quantification is mentioned in lines 101-111.

Mention vector background for overexpression construct

The vector background is mentioned in line 129.

Generation at which the transgenic lines were analysed

It is mentioned in line 136.

Line 151 the word dessication should be replaced by dehydration or drought

The dessication word is replaced by dehydration in the manuscript.

FIG 2C the legend mentions scale bar as 1 cm however the picture does not appear to be in same scale, hence should be otherwise removed

Although the scale bar represents 1cm, their lengths are different in both images, i.e. the WT’s scale bar of 1cm has an actual size of 0.489cm whereas the one for transgenic plants is 0.351cm.
